# An Approach to Presacral Mass in Neonates

**Published:** 2014-04-01

**Authors:** Bilal Mirza, Muhammad Saleem

**Affiliations:** Department of Pediatric Surgery, The Children’s Hospital and the Institute of Child Health Lahore, Pakistan

**Dear Sir**

Cystic masses in presacral space in a neonate have a number of differential diagnoses including type-IV sacrococcygeal teratoma (SCT), pelvic neuroblastoma, anterior meningocele, and rectal duplication cyst.[1-6] Every lesion has certain specific features that may give an insight to the preoperative diagnosis and help in individualizing the surgical approach as to the type of lesion. In case of sacrococcygeal teratoma a coccygectomy has to be added in the surgical therapy to reduce the chance of recurrence.[5] The presentation of a presacral mass is usually independent of the type of the lesion. It may compress the rectum, ureters or bladder neck depending upon the size and consistency of the lesion. The result can be constipation, abdominal distension, urinary outlet obstruction or hydroureteronephrosis etc.[1-5] The mass may be palpable in the lower abdomen. Digital rectal examination with little finger may aid in the confirmation of the presacral location of the mass. X-ray abdomen may show calcifications in the mass. Ultrasound of the lesion may provide an information regarding consistency of the lesion any calcifications and relation with the surrounding structures. An ultrasound of the spine may also help to detect any spinal dysraphism to rule out anterior meningocele.[6] CT or MRI is of more importance as they give clearer picture about consistency, relation with the surrounding structures, and calcifications. Calcifications are important as its presence alerts the surgeon about the nature of the lesion. 

Two most important lesions that may have calcifications are type-IV SCT and neuroblastoma; but, the surgical approach to both of these lesions is different as SCT requires coccygectomy in addition to the mass excision through another incision on the buttock. On the other hand, coccygectomy and another incision are avoidable in case of neuroblastoma and other lesions at the same location. Therefore, further work including tumor markers like alpha fetoprotein (AFP) or β-HCG should be performed and interpreted with reference to the age of neonate as AFP level may be in thousands is normal in neonatal age.[4,5] The surgical approach for a rectal duplication, type-IV SCT, and neuroblastoma is initially similar, i.e., initial mobilization is done through a transverse lower abdomen incision. If the mass is cystic and external appearance is that of a bowel in intimate contact with the rectum it may be a case of rectal duplication cyst. In this case, surgeon may decide to completely excise it with additional posterior sagittal, trans-anal or trans-coccygeal approaches or excision of as much as portion with mucosal stripping of the rest through the same incision.[1-3] We had a 15-day-old male neonate presented with constipation and abdominal distension; ultrasound showed a cystic mass in the pelvis causing bilateral hydroureteronephrosis. CT scan also showed a cystic mass without calcification. AFP was in normal range. At operation, a gut like mass was present posterior to the rectum and intimately attached to it (Fig. 1). The mass was opened and mucoid secretions drained. The most part of the mass removed followed by mucosal stripping of the rest. Postoperative recovery was uneventful. Histopathology confirmed the mass a rectal duplication cyst. There is no recurrence at 1 year follow-up.

**Figure:**
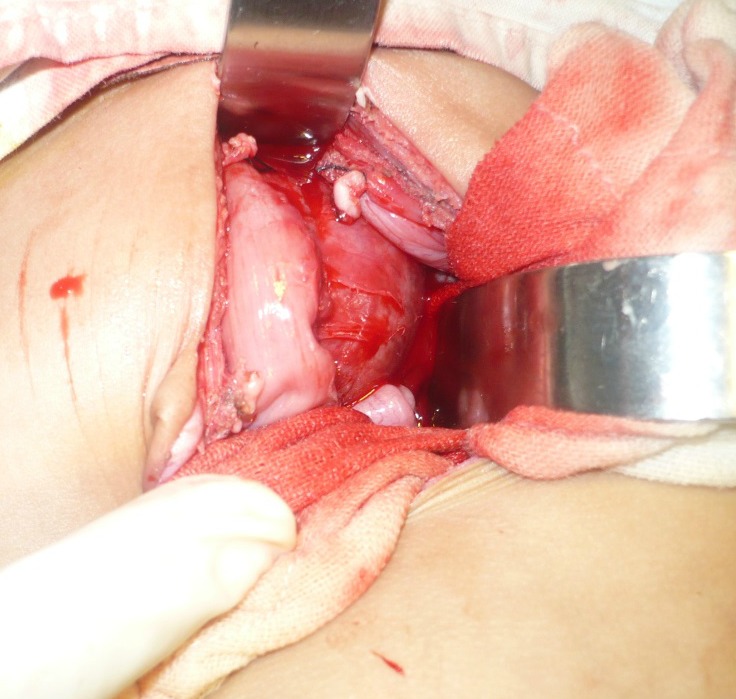
Figure 1: Rectal duplication cyst

In some cases with calcifications in the mass, the differentials are further converged to sacrococcygeal teratoma and rarely presacral neuroblastoma.[4.5] Problem arises when AFP is inconclusive of SCT. Raveenthiran [5] called SCT as great masquerader. However, sometimes the inverse may be true. We had a 25-days-old male baby presented with constipation and a palpable mass in the pelvis. Ultrasound and CT scan showed calcifications in the mass. The CT scan opinion was type-IV SCT (Fig. 2). The patient was explored from lower abdominal incision. The mass was dissected retro-rectally and surprisingly entire mass was excised in-toto attached to a nerve fiber (Fig. 3). It was decided to postpone the coccygectomy till the biopsy confirmation as the mass was firm (less suspicious of SCT in setting of normal AFP) and attached to a nerve. Biopsy was suggestive of neuroblastoma. 

**Figure:**
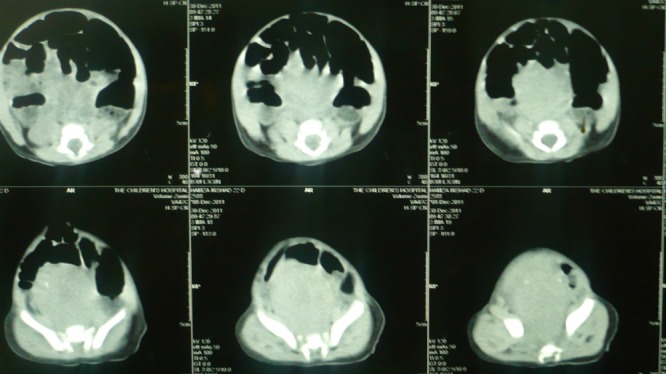
Figure 2: A presacral mass with calcifications.

**Figure:**
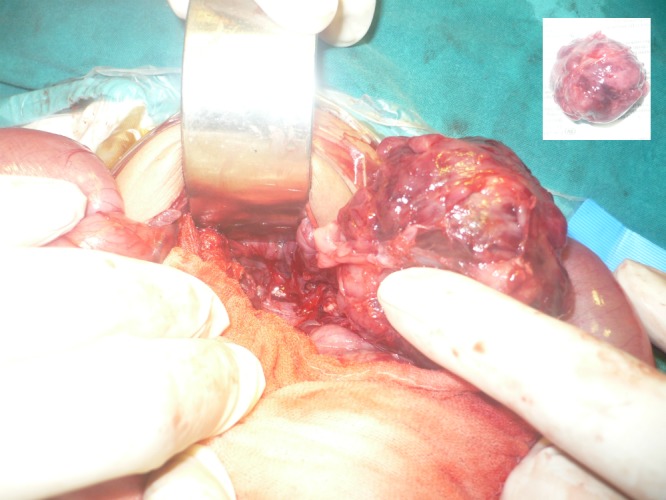
Figure 3: Presacral neuroblastoma. Inset shows complete excision.

## Footnotes

**Source of Support:** Nil

**Conflict of Interest:** The author is an Editor of the journal. But he did not take part in the evaluation or decision making of this manuscript. The manuscript has been independently handled by two other editors.

